# Colloidal stability of phytosynthesised gold nanoparticles and their catalytic effects for nerve agent degradation

**DOI:** 10.1038/s41598-021-83460-1

**Published:** 2021-02-18

**Authors:** Veronika Holišová, Martin Urban, Zuzana Konvičková, Marek Kolenčík, Pavel Mančík, Jiří Slabotinský, Gabriela Kratošová, Daniela Plachá

**Affiliations:** 1 Nanotechnology Centre, CEET, VŠB – Technical University of Ostrava, 17. listopadu 2172/15, 708 00 Ostrava, Czech Republic; 2 National Institute for Nuclear, Biological and Chemical Protection, v.v.i., Kamenná 71, 262 31 Milín, Czech Republic; 3 ENET Centre, CEET, VŠB – Technical University of Ostrava, 17. listopadu 2172/15, 708 00 Ostrava, Czech Republic; 4 Department of Soil Science and Geology, Slovak University of Agriculture in Nitra, Tr. A. Hlinku 2, 949 76 Nitra, Slovak Republic

**Keywords:** Colloids, Soft materials, Nanoscale materials, Nanoparticles

## Abstract

Herein, *Tilia* sp. bract leachate was used as the reducing agent for Au nanoparticles (Au NPs) phytosynthesis. The colloidal properties of the prepared Au NPs were determined to confirm their stability over time, and the NPs were then used as active catalysts in soman nerve agent degradation. The Au NPs characterisation, reproducibility and stability studies were performed under transmission electron microscopy**,** ultraviolet visible spectroscopy and with ζ-potential measurements. The reaction kinetics was detected by gas chromatography coupled with mass spectrometry detector and solid-phase micro-extraction to confirm the Au NPs applicability in soman hydrolysis. The ‘green’ phytosynthetic formation of colloidal crystalline Au NPs with dominant quasi-spherical shape and 55 ± 10 nm diameter was successfully achieved, and there were no significant differences in morphology, ζ-potential or absorbance values observed during the 5-week period. This verified the prepared colloids’ long-term stability. The soman nerve agent was degraded to non-toxic substances within 24 h, with 0.2156 h^−1^ reaction rate constant. These results confirmed bio-nanotechnology’s great potential in preparation of stable and functional nanocatalysts for degradation of hazardous substances, including chemical warfare agents.

## Introduction

Chemical warfare agent (CWA) decontamination has a very high priority in military defence and especially in the current fight against terrorism. The Soman (O-Pinacolyl methyl-phosphonofluoridate) examined in this article is a CWA with extreme toxicity for biota. This organophosphate is a nerve agent (NA) which compromises the normal nervous system functioning by inhibiting acetylcholinesterase catalytic breakdown of acetylcholine and other choline ester neurotransmitters^1,2^. Soman causes death within a few minutes to a few hours after exposure, dependent on the dose and route of exposure, but it can be degraded by hydrolytic cleavage of its P–F bonding^1^. Some toxicology studies show that pinacolylmethylphosphonic acid (PMPA) is formed as an intermediate substrate and that the methylphosphonic acid (MPA) usually formed as a final degradation product of soman hydrolysis is considered non-toxic^2^. Several studies have also focused on the degradation of CWAs and their simulants using different kinds of NPs. The Fe, Zn and Al metal nano-dispersed oxides or oxo-hydroxides have been prepared by homogeneous hydrolysis of sulphates, nitrates and chlorides and tested for their ability to convert nerve-agents to non-toxic products at 25 °C^3^.

Chemical preparation of Au NPs supported on mesoporous TiO_2_ achieved effective soman photocatalytic decontamination^4^. The nanocomposite was prepared by biosynthesis using the *Mallomonas kalinae* brown algae with SiO_2_ on its surface^5^, and soman degradation was confirmed by the nanogold embedded on this surface. Nanogold is a well-known catalytic nanomaterial, and Au NPs exhibit the thermodynamic stability, inertness, electric and optic conductivity applicable in a wide variety of catalysis. Au NP’s catalysis is generally possible because of their large area and decreasing particle size distribution^6–8^, and the catalytic effect is significantly enhanced at higher nanogold crystallinity, by certain morphology types and uncovered grain boundaries. In addition, collective oscillation of conductive electrons between the dielectric and metal–surface plasmon resonance (SPR) occurs under an external optical field^6,7^, and the combination of these characteristics can lead to the disruption of active bonds and groups of atoms in the organophosphate molecule^9^. Most importantly, Au NPs have no discernible toxic effects on living organisms^10^.

NPs can be prepared by biosynthesis as well as chemical and physical methods, and this provides an ecological approach of bottom-up synthesis^11^ where inorganic cores with functional organic packaging are formed. Relatively long-term stability and variability over a wide application range have recently proved surprising^11^, and Au NP biosynthesis has been confirmed in bacteria^12^, cyanobacteria^13^, algae^8^, microscopic fungi^14^ and different plant extracts^15–17^. The current use of plants or plant parts leachates and/or extracts for NPs biosynthesis is simple and attractive. This phytosynthetic method uses soluble plant substances such as alkaloids or simple phenolic compounds which possess both reduction and stabilisation effects^17,18^.

NPs stability is one of the most important properties in nanomaterial application^15,16,19^, and this is based on: (i) NP size, surface to volume ratio, crystallinity and morphology^20^; (ii) NP exposed surface charged with specific ions and an electric double-layer structure with specific capacity^21^ and (iii) the ionic strength and pH, and other critical factors including temperature^21^.

Nanogold’s remarkable properties make it one of the most catalytically active elements. Stable biosynthesised spherical Au NPs anchored on a silica surface have been employed in CO conversion^8^, and phytosynthesised nanogold was used for nitrophenol and organic dye degradation in the presence of NaBH_4_^11,19^. These effects inspired us to conduct the Au NPs phytosynthesis with *Tilia* sp. linden bract aqueous leachate in order to verify the NPs stability and experiment reproducibility. Finally, the Au NPs were used for soman degradation with subsequent evaluation by gas chromatography coupled with mass spectrometry and solid-phase micro-extraction.

## Results and discussion

### UV–VIS measurement

**Figure 1:**
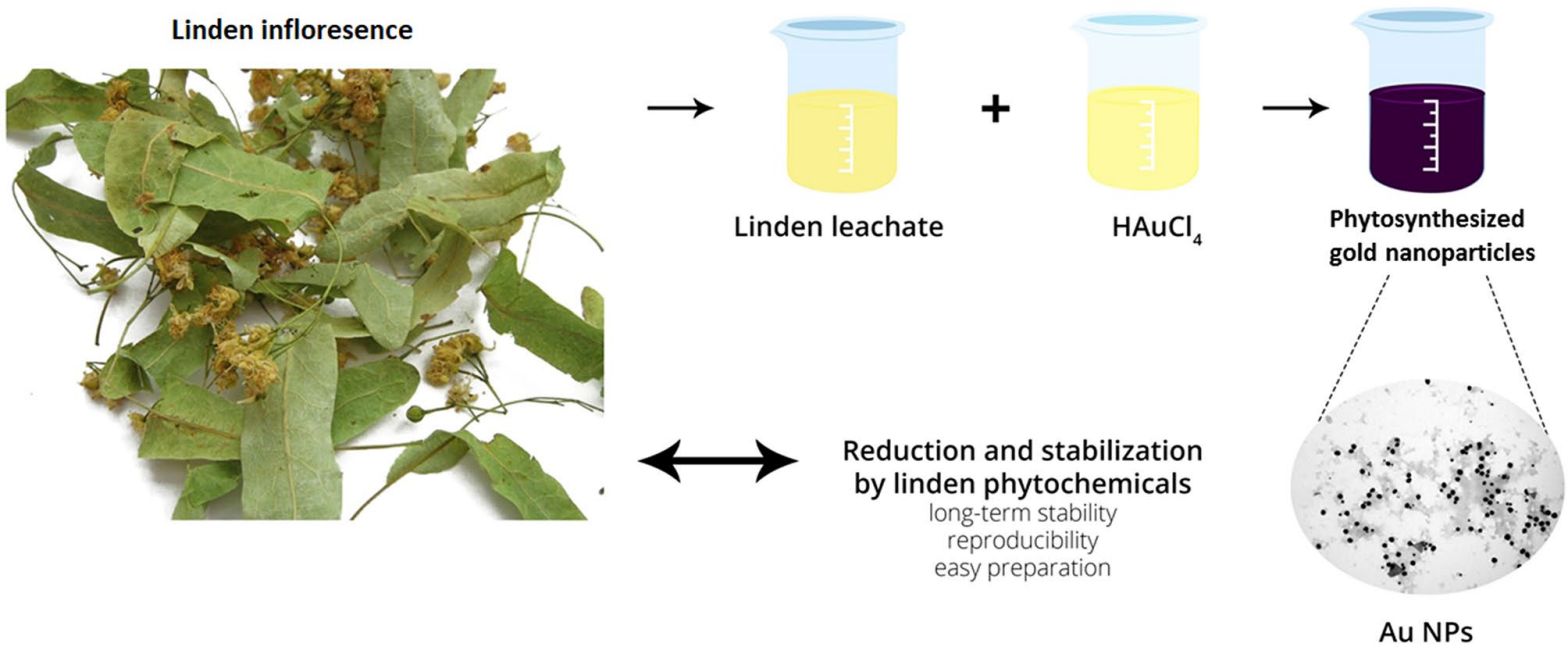
Batch phytosynthesis of Au NPs using linden bracts leachate.

Suspension colour change from yellow to dark purple was observed after mixing the Au precursor and plant leachate for 15 min (Fig. 1). The nanogold batch phytosynthesis in all Au1-Au5 samples indicates the linden bracts leachate reducing potential, where the Au(III) ions are reduced to Au^0^ by phytochemicals present in the leachate^11^. Studies suggest that biomolecules such as proteins, enzymes and flavonoids can reduce Au(III) ions during phytosynthesis to form Au NPs and stabilise them directly in a one-step process^22,23^.

**Figure 2:**
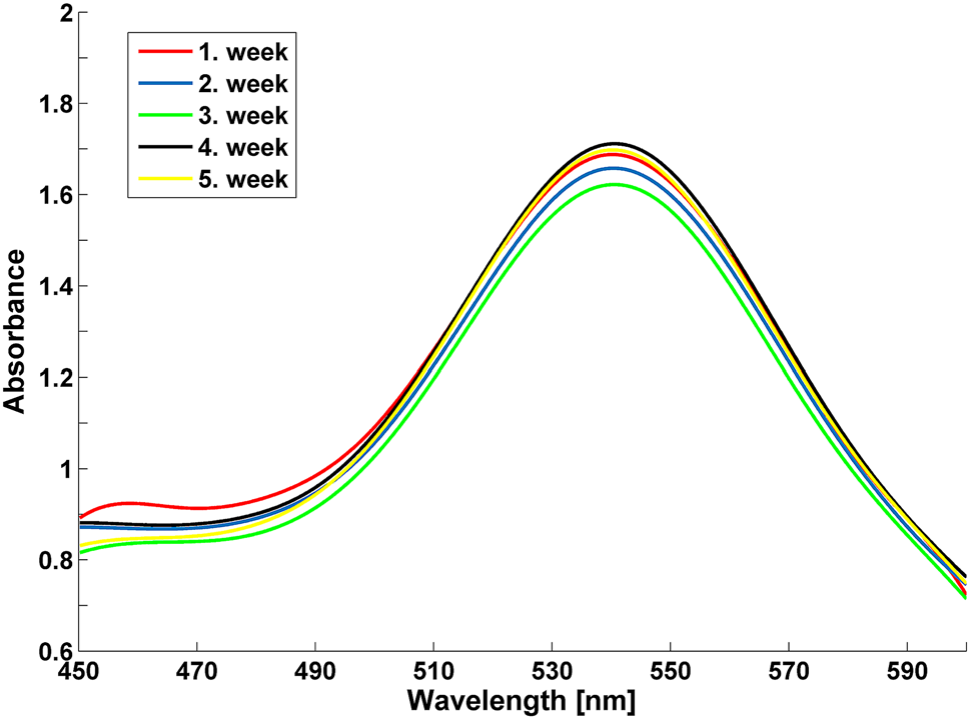
UV–VIS spectra of Au1 sample measured over 5 weeks.

The Au samples’ absorption spectra were measured in the 490 to 600 nm range and the characteristic absorption peaks were regularly determined over 5 weeks. The linden leachate and Au precursor mixtures exhibited one absorbance peak at 540–548 nm in the Au1-Au5 samples. This identified the Au NPs’ SPR phenomenon^24^. Figure 2 depicts the Au1 sample which was selected for soman degradation testing as a representative for all analyses.

NPs UV–VIS absorption is mainly influenced by size, shape, concentration, agglomeration capacity and the refractive index of the NP surface^20,25^. If NPs destabilise over time, the original absorption peak will decrease in intensity due to depletion of stable NPs. The peak is then broadened, or a secondary peak is formed at longer wavelength because of the formation of aggregates or agglomerates. Their formation also leads to changes in band position or evolution of a new SPR peak at higher wavelength^20^.

There was no change in the absorption maxima wavelength (λ_max_) observed for the Au1 sample at 540 nm over the 5-week experiment (Fig. 2). Intensity did not change significantly during measurement, and no NP aggregation occurred in this period. Figure 2 also enables the prediction that the colloid contains NPs with a wider distribution of particle size and shape^20,25^. Table 1 presents the arithmetic mean and standard deviation of absorption intensity (A) and absorbance maxima (λ_max_) for the five Au NPs preparation repetitions.

**Table 1:** The arithmetic mean and standard deviation of absorption intensity (A) and absorbance maxima λ_max_ Au colloids prepared in parallel.

Week	A	λ_max_ [nm]
1	1.63 ± 0.15	543.40 ± 3.13
2	1.60 ± 0.12	543.20 ± 3.27
3	1.57 ± 0.16	542.40 ± 3.29
4	1.70 ± 0.10	542.40 ± 3.21
5	1.66 ± 0.11	543.20 ± 3.27

The UV–VIS spectroscopy generally confirmed minor changes in absorbance and absorption maxima of the Au1-Au5 samples during the experimental period (Table 1) and the Au NPs in the colloid dispersion were stable. Moreover, no significant aggregation was observed in any prepared colloid and no visible colloid colour change occurred during storage. These results are further strengthened by the ζ-potential measurements.

### Size and morphology of phytosynthesised Au NPs

**Figure 3:**
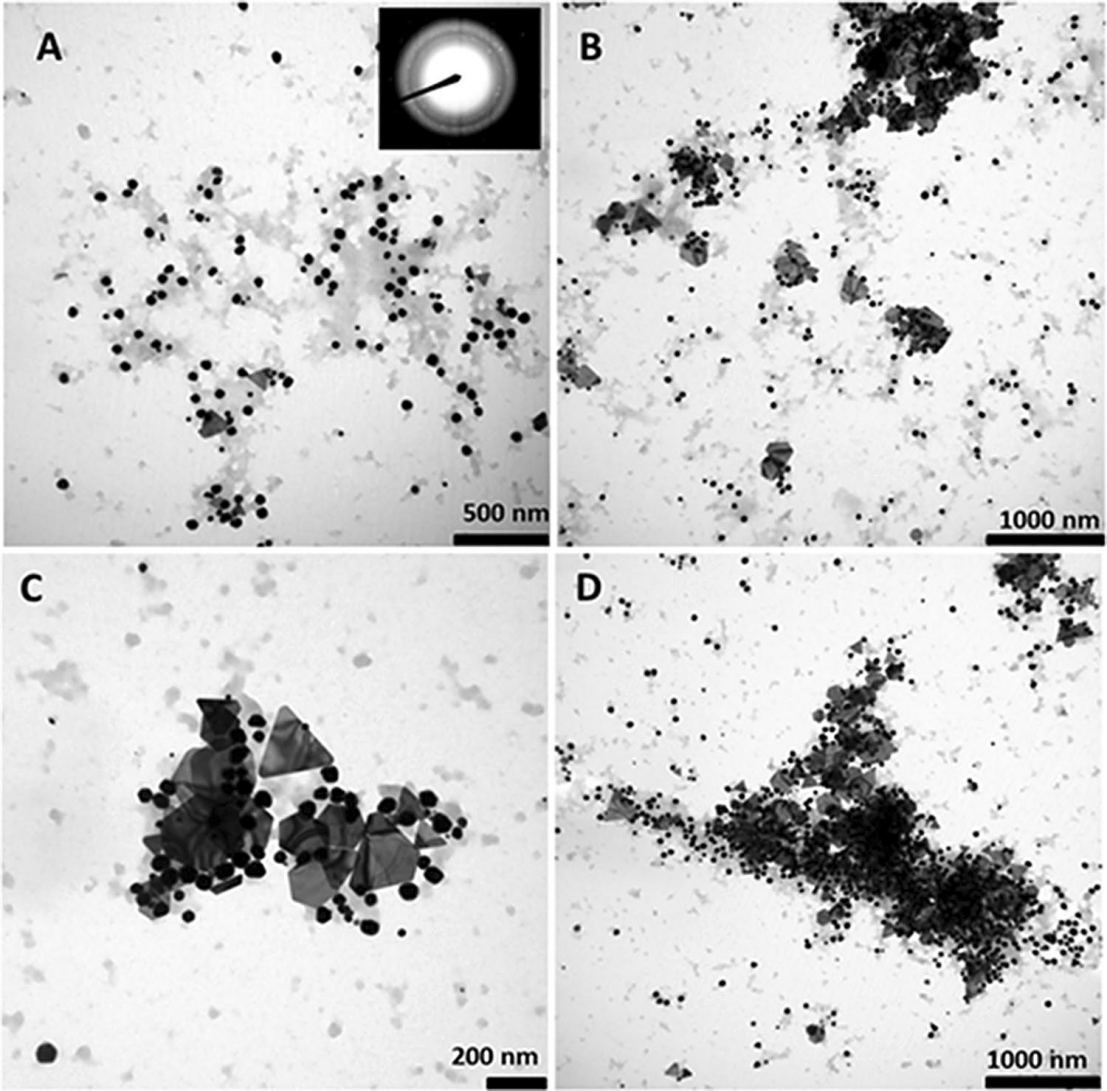
TEM analysis reveals phytosynthesised Au1 NPs shape and size heterogeneity. Crystalline Au NPs were confirmed by SAED. (**A**) The majority are quasi-spherical with some presence of triangular (**B**) and hexagonal (**C**) particles evident. Au NPs were detected both isolated and in groups (**D**).

The size, morphology, and crystal structure of synthetised Au NPs were characterised by TEM. Figure 3 shows their varied shape in the illustrated Au1 sample which has quasi-spherical and triangular nanoplates and hexagonal Au NPs. The Au1 sample size of quasi-spherical NPs was 51 ± 11 nm, and 184 ± 46 nm for triangular and hexagonal nanoplates. No significant changes in NP size were observed in these parameters within 5 weeks; with 46 ± 14 nm and 180 ± 50 nm observed in the 5th week. There was also a more stabilised and less contrasting phytochemical coating around the NPs.

Statistical analysis of the Au1-5 samples established that the dominant NP shape was quasi-spherical with average 55 ± 10 nm size, and the triangular and hexagonal-shaped Au NPs’ size distribution was 199 ± 55 nm. The mean quasi-spherical and non-spherical NP sizes were evaluated 5 weeks after initial observation, with the following results; quasi-spherical NPs measured 55 ± 12 nm and non-spherical NPs 194 ± 54 nm.

Previous UV–VIS spectra provided no evidence of different absorption peaks for smaller and larger Au NPs. This may have been due to the higher concentration of Au NPs with size around 60 nm which corresponds to absorption maxima of approximately 540 nm^26^. However, TEM characterisation confirmed the presence of NPs crystalline structure with broader particle size and shape distribution.

While our phytosynthetic method has established reproducible results, minor differences are noticeable in the observed samples. This is most likely due to the amount and content of phytochemicals in the applied plant biomass. However, further imposed conditions, such as the source of bio-reductants and bio-stabilisers, the type of metal precursor and its concentration and the contact time between biomass and precursor may enhance the reproducibility of Au NPs production with green synthesis emphasis.

In addition, the formation of Au NPs with different sizes and shapes herein was most likely associated with the wide range of biomolecules in linden bract leachate such as gallic acid, catechin and quercetin^27,28^. For example, the quercetin presence could be responsible for formation of spherical Au NPs in the size range from 20 to 45 nm^16^. Choi et al. and Gavade et al. also described the biosynthesis and stabilisation of spherical nanoparticles and triangular and hexagonal Au NPs with the size range from 17 to 80 nm mediated by catechin and gallic acid^22,29^. In addition, the linden bracts in our experiments contain the previously mentioned phytochemicals which are approved as suitable biomass for Au NP reduction and stabilisation^30,31^.

### ζ-potential measurement

The ζ-potential denotes a double-layer electrostatic surface potential which is highly dependent on the immediate environment, and its value adequately determines NPs stability. The conventional theoretical boundary between NP stability and instability lies between − 30 and + 30 mV; and lower and higher values than these boundaries establish colloidal stability status^21^. Table 2 herein highlights the established moderately stable Au1 colloidal pH and ζ-potential values.

**Table 2:** ζ-potential and pH values of Au1 samples over the five-week experimental period.

Week	ζ –potential [mV]	pH
1	− 24.1 ± 0.5	2.19
2	− 21.9 ± 1.7	2.15
3	− 20.8 ± 0.6	1.77
4	− 20.2 ± 1.4	2.03
5	− 19.9 ± 1.4	2.01

**Figure 4:**
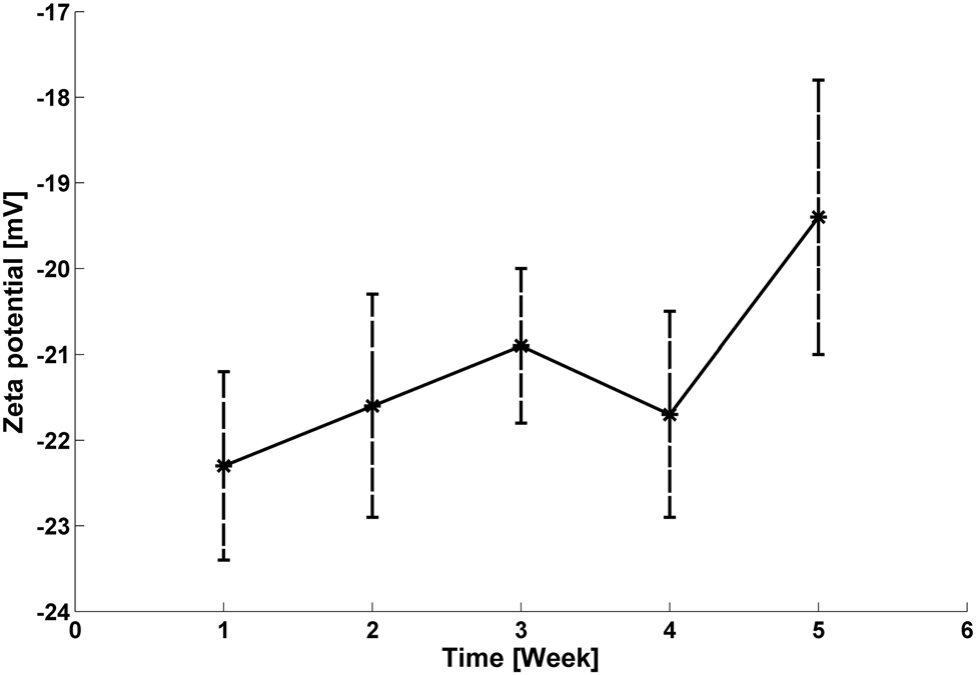
Arithmetic mean and standard deviation of ζ-potential values for the measured samples, including Au1.

The ζ-potential was regularly measured for all samples, and Fig. 4 shows the slight alteration in ζ-potential arithmetic mean over the experimental period. The phytosynthesised Au NPs were classified as moderately stable because the measured values did not reach the estimated stability boundary. The negative ζ-potential values were detected here because the capping agents comprised active bioorganic compounds with long-term change effects^32^. We then considered the stabilisation process complete because no significant change was noted in ζ-potential value.

**Figure 5:**
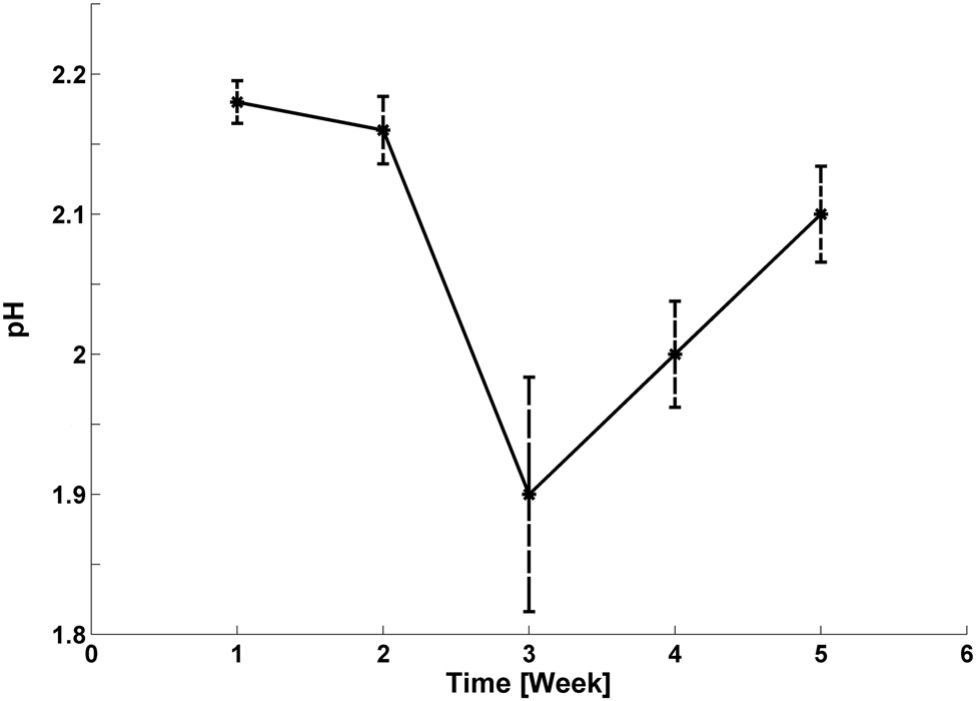
Arithmetic mean and standard deviation of pH values for measured samples including Au1.

The Au NPs samples’ pH value was continuously monitored because the ζ-potential value fundamentally depends on pH and temperature change. Figure 5 herein shows that the pH of all Au NPs samples was constant throughout the experimental period at approximately pH 2 at constant laboratory temperature.

### Soman degradation

Hydrolytic degradation of soman was performed in the presence of three materials: (i) pure linden bracts leachate (C1); (ii) HAuCl_4_ Au precursor and (iii) colloidal Au NPs (Au1).

**Figure 6:**
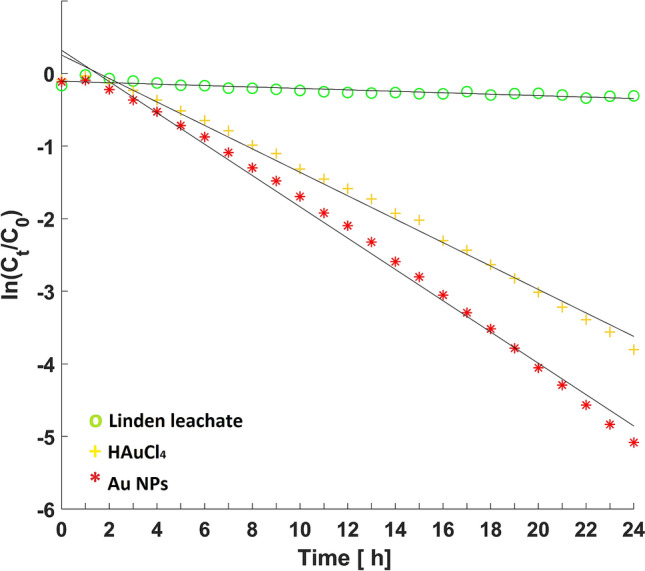
Kinetic degradation of soman hydrolysis in the presence of linden leachate, gold precursor and Au NPs.

Figure 6 shows the hydrolysis course catalysed by colloidal Au NPs with 2.5 mmol .dm^−3^ Au compared to soman degradation with Au precursor containing the same Au concentration and the Cl control sample. The Au NPs achieved 99.38% soman conversion in 24 h with 0.2156 h^−1^ degradation rate constant, and the phytosynthesised Au NPs can influence soman hydrolysis through the quantum-size effects generated by electrons confined in a small volume. The literature states that a basic environment of pH 8–14 shifts the reaction equilibrium to degradation products. However, the Au NP catalytic effect has been confirmed in the pH 2.2 acidic environment, and this is much better than literature reports of hydrolysis proceeding at neutral pH^5^.

The resultant conversion of soman in the presence of the pH 4 Au precursor was 97.77% after 24 h with attendant reaction rate constant of 0.1614 h^−1^. The differing Au(I) and Au(III)) oxidation states participate as catalysts in organic synthesis. For example, the HAuCl_4_ Au precursor used as a catalyst degraded soman due to its oxidation potential^33^.

Although the pH 5.1 linden leachate exhibited no degradation effect on the soman nerve agent and the phytochemicals in the water extract did not influence soman decomposition, soman conversion by linden leachate proceeded in a similar manner to the simple soman hydrolysis^5^.

The degradation products were determined by SPME and GC/MS screening analysis. The amount of MPA standard solution, as the final degradation product, was 100 ng, and Table 3 highlights identification of the following compounds by mass spectra: ethylacetate (Et-Ac, CAS: 141-78-6), pinacolylalcohol (P-ol, CAS: 464-07-3), dipinacolylmethyl phosphonate (DPMP, CAS: 7040-58-6), soman (CAS: 96-64-0).

**Table 3:** SPME monitoring of hydrolysis products after 24 h.

Samples	Monitored analytes (Peak area)			
	Et-Ac	P-ol	DPMP	Soman
Linden leachate	–	688, 457	1,345,668	38,689,503
HAuCl_4_ (2.5 mmol·dm^−3^)	847,594	1,719,116	5,953,960	1,297,049
Au NPs (2.5 mmol·dm^−3^)	–	1,995,211	1,043,776	336,166

**Figure 7:**
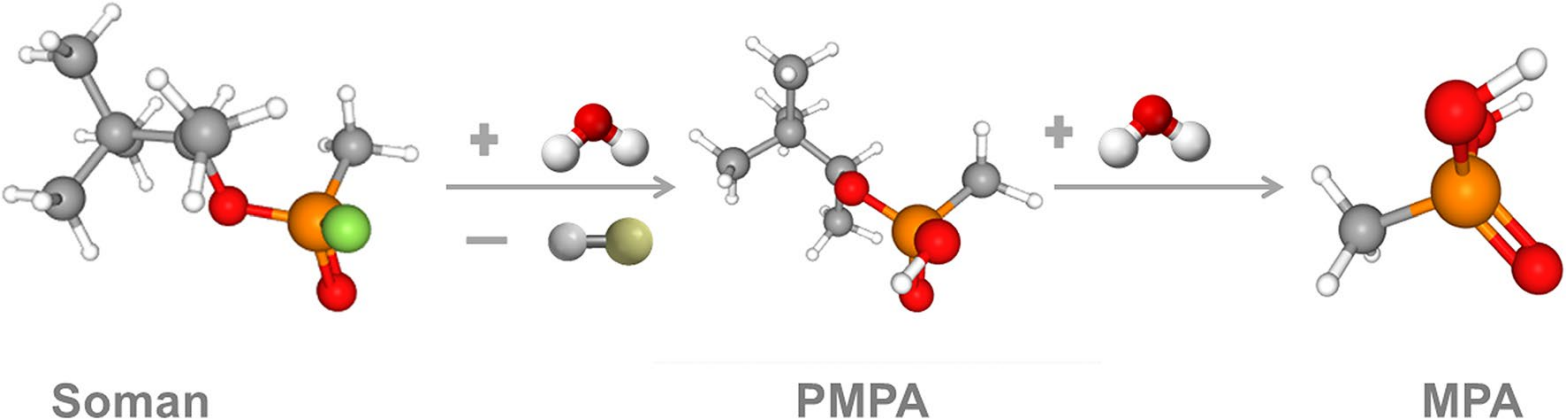
Hydrolysis of soman^34,35^.

The following were also present in the mass spectrum (1) Si-pinacolylmethylphosphonic acid PMPA—the Si derivative formed after hydrolysis of the P-F bond and (2) Si-methyl phosphonic acid MPA—the Si derivative formed after hydrolysis of the P-F and P-O bonds. Figure 7 shows that PMPA was the product most formed in the first stage of the samples’ soman hydrolysis, and Table 4 depicts that MPA was detected as a final non-toxic degradation product^34,35^.

**Table 4:** Products of soman degradation after 24 h detected by GC/MS screening analyses.

Samples	Monitored analytes (Peak area)		
	Soman	PMPA-Si	MPA-Si
Standard solution of soman (100 ng)	11,391,354	–	–
Standard solution of MPA-Si (100 ng)	–	–	44,622,216
Linden leachate	35,872,335	738,678	745,057
HAuCl_4_ (2.5 mmol·dm^−3^)	–	1,874,961	134,487
Au NPs (2.5 mmol·dm^−3^)	–	23,369,827	501,546

Soman hydrolysis was catalysed more effectively with Au NPs than with HAuCl_4_ Au(III) ions. The soman degradation mechanism was most likely different in the presence of Au(III) ions to the Au NPs action. We observed that ethyl acetate and the highest concentration of DPMP were detected in the sample only when HAuCl_4_ was present, because the PMPA-OH group was then preferentially esterified to form DPMP. Therefore, the concentration of the low-molecular PMPA and MPA final products in the Au NPs’ sample was 12.5 and 3.7 times higher, respectively, than in the reaction where HAuCl_4_ was mixed with soman.

Finally, although samples with nanogold had lower pH value, the Au NPs provided better catalytic activity for soman hydrolysis than HAuCl_4_ after 24 h. In addition, toxicology studies record that pinacolylmethylphosphonic acid is formed as an intermediate and methylphosphonic acid is usually formed as a final degradation product of soman hydrolysis and is considered non-toxic^2^.

## Methods

### Au nanoparticle phytosynthesis

*Tilia sp.* bracts were collected in the Nová Ves area of Frýdlant nad Ostravicí in The Czech Republic. Here, 2 g of the dried biomass and 50 mL of 80 °C Milli-Q water were mixed for 15 min at static condition. The leachate was filtered through a 0.22 µm syringe filter (MCE, Millex-GS, Merck Millipore, Germany) and 5 mmol·dm^−3^ aqueous solution of HAuCl_4_ (Sigma-Aldrich, USA) was used as initial precursor for AuNP generation. The leachate and precursor were mixed in 1:1 v/v ratio for 15 min and the final colloid was maintained in the dark at 5 °C.

The Au NPs and linden bracts leachates preparations were repeated five times to control phytosynthetic reproducibility. The final AuNP samples were denoted Au1, Au2, Au3, Au4 and Au5, and the C1, C2, C3, C4 and C5 linden bracts leachates of each sample were stored as controls. Finally, the colloidal solutions were brought to 25 °C for subsequent experiments and measurements.

### Physical–chemical properties of colloidal Au nanoparticles

#### Study of Au colloidal stability and phytosynthetic reproducibility

Au NP stability and phytosynthesis reproducibility were studied by regularly measuring UV–VIS absorption maxima, ζ-potential and colloid pH values each week for 5 weeks for all Au1-Au5 samples. The characteristic Au NPs absorption peaks were measured by UV–VIS spectrophotometer (LAMBDA 11, Perkin Elmer Instruments, USA) with 0.5 nm unit step in the 450-600 nm wavelength range. The ζ-potential was periodically controlled by ZetaSizer Nano–ZS (ZEN 3600; Malvern Instruments Ltd., UK), and solutions were evaluated on system acid–base equilibrium by measuring pH values by EUTECH pH 5 + meter (Eutech Instruments, USA).

#### NPs size distribution, morphology and crystal structure determination

NP morphology and size distribution were characterised by transmission electron microscopy (TEM) at 80 kV using JEOL 1200 EX (JEOL, Japan), and the crystal structure was monitored by selected area electron diffraction (SAED). The 2μL liquid sample was placed on a copper grid coated with carbon and dried under laboratory conditions, and the NPs size distribution was evaluated by JMicroVision programme with approximately 150 NPs analysed per sample. (www.jmicrovision.com).

### Soman degradation

The soman degradation experiments were performed at the National Institute for Nuclear, Biological and Chemical Protection at Kamenná in The Czech Republic. The GC–MS system (GC7890A/MSD5975 C, inert XL, Agilent Technologies, USA) equipped with automatic solid phase micro-extraction (CTC PAL, Thermo Scientific, USA) and HP-5MS silica column (30 m × 0.25 mm × 0.25 µm film thickness) monitored soman degradation. Solid phase micro-extraction (SPME) device with 65-micron polydimethylsiloxane/divinylbenzene (PDMS/DVB) Stableflex Supelco fibre was utilised for soman transfer and injection into the GC injection port. Both sorption and consequent desorption time was 300 s in both cases at 30 °C and 250 °C, respectively. Agitation at 500 rpm for 180 s then homogenised the sample with the following GC temperature program was as follows: 45 °C (1 min), 15 °C/min, 80 °C (1 min), 25 °C /min up to 280 °C (5 min). The 99.9% pure Helium carrier gas had 1 mL min^−1^ flow rate, and the degradation products were detected and determined utilising MSD ChemStation software E.02.02.1431 with the NIST 08 library of mass spectra.

Three different samples were tested for soman degradation. Their injections into the GC column was performed at hourly intervals for 24 h as follows: 5 µl of soman (purity > 95%, concentration in the resulting solution was 341 µg cm^−3^) was mixed with 15 ml of (i) linden leachate C1 as the control experiment, (ii) HAuCl_4_ precursor (2.5 mmol dm^−3^ of Au) and (iii) Au1 an aqueous solution of linden leachate containing Au NPs (2.5 mmol dm^−3^ of Au). Residual concentrations of soman after degradation monitored for 24 h were fitted using pseudo-first order kinetics and the rate constant (*k*) was calculated by Eq. (1):

1 $${\mathrm{ln}(c}_{t}{/c}_{0})=-k\mathrm{t}$$

where c and c_0_ describe the percentage content of soman at given time *t* and time zero, respectively, and *t* is reaction time (h). Soman conversion was then calculated by Eq. (2).

2 $$X_{{{\text{soman}}}} = {\text{c}}_{0} - {\text{ c}}_{{\text{t}}}$$

where *X*_soman_ is the percentage of soman conversion and c_t_ percentage is the content of soman in time *t*. The degradation of soman using sample Au1 was repeated two times.

#### Analysis of degradation products

Decreased soman concentration over time was observed in the samples described above. All three samples were individually extracted after 24-h soman degradation into 2 × 3 mL CH_2_Cl_2_/MeOH (9:1) for determination of formed degradation products. The organic extracts were dried by Na_2_SO_4_ and 1 mL aliquots of each extracts were derivatised by adding 25µL of BSTFA derivative agent (N,O-Bis (trimethylsilyl) trifluoroacetamide). The reaction mixture was shaken by hand and this was followed by derivatisation for 2 h at 40–50 °C. Finally, 1µL was taken from each reaction mixture for GC/MS screening analysis and mass spectra were evaluated by NIST08 database.

### Statistical analysis

All graphs were created in MATLAB software (MathWorks, USA). The arithmetic mean and standard deviation of UV–VIS data, NPs size, ζ-potential and the pH values of Au1-Au5 colloids were processed by standard formulae in MATLAB software (MathWorks, USA).

## Conclusions

Colloidal nanoparticle properties ensure that they are very promising catalytic agents. However, in addition to the necessity that NPs preparation methods and techniques are both economically and environmentally sustainable, increasing attention in industrial chemistry now focuses on ‘green chemistry’ where synthesis is inspired by processes closer to nature. Therefore, our Au NPs were prepared and stabilised by eco-friendly phytosynthesis.

Linden bracts water leachate was used as the reducing and capping agent, and the preparation of Au NPs was repeated five times to control phytosynthesis reproducibility. Here, the phytosynthesised Au colloid ζ-potential values moved around.

-20 mV with approximately 540 nm Au NP absorption maxima. The average Au NP sizes were 55 ± 10 nm for quasi-spherical NPs and 199 ± 55 nm for polygonals. Importantly, the phytosynthesised NPs preparation method was proven reproducible, and acceptable colloid was confirmed in the 5-week period.

In addition, the phytosynthesised Au NPs’ catalytic activity was established by soman degradation within 24 h. While it was interesting to note that the HAuCl_4_ Au NPs precursor also proved able to degrade soman, the Au NPs provided much better catalytic activity. This was due to the previously mentioned peak area of the PMPA and MPA low-molecular final products in the sample, with the Au NPs being 12.5 and 3.7 times higher, respectively, than soman degradation induced solely by HAuCl_4_.

In conclusion, this research confirmed the high potential of bio-nanotechnology for reproducible preparation of stable and functional nanocatalysts for degradation of hazardous substances, and most importantly, these catalytically active nanomaterials can be prepared by green biotechnology.
